# Post‐Myocardial Infarction Remodeling and Hyperkinetic Remote Myocardium in Sheep Measured by Cardiac MRI Feature Tracking

**DOI:** 10.1002/jmri.29496

**Published:** 2024-06-28

**Authors:** Steven K.S. Cho, Jack R.T. Darby, Georgia K. Williams, Stacey L. Holman, Archana Rai, Joshua F.P. Van Amerom, Chun‐Po Fan, Christopher K. Macgowan, Joseph B. Selvanayagam, Janna L. Morrison, Mike Seed

**Affiliations:** ^1^ Department of Physiology, Faculty of Medicine University of Toronto Toronto Ontario Canada; ^2^ Early Origins of Adult Health Research Group, Health and Biomedical Innovation, UniSA: Clinical and Health Sciences University of South Australia Adelaide South Australia Australia; ^3^ Preclinical, Imaging & Research Laboratories South Australian Health & Medical Research Institute Adelaide South Australia Australia; ^4^ Division of Cardiology The Hospital for Sick Children Toronto Ontario Canada; ^5^ Department of Medical Imaging, University Medical Imaging Toronto University of Toronto Toronto Ontario Canada; ^6^ Peter Munk Cardiac Center Toronto General Hospital, University Health Network, University of Toronto Toronto Ontario Canada; ^7^ Research Institute The Hospital for Sick Children Toronto Ontario Canada; ^8^ Rogers Computational Program, Ted Rogers Centre for Heart Research, Peter Munk Cardiac Centre, University Health Network University of Toronto Toronto Ontario Canada; ^9^ Department of Medical Biophysics, Faculty of Medicine University of Toronto Toronto Ontario Canada; ^10^ Cardiac Imaging Research Group, Department of Heart Health, South Australian Health and Medical Research Institute Flinders University Adelaide South Australia Australia

**Keywords:** Animal model, Feature tracking, Late gadolinium enhancement, Myocardial infarction, Myocardial strain, Sheep

## Abstract

**Background:**

Cardiac MRI feature tracking (FT) allows objective assessment of segmental left ventricular (LV) function following a myocardial infarction (MI), but its utilization in sheep, where interventions can be tested, is lacking.

**Purpose:**

To apply and validate FT in a sheep model of MI and describe post‐MI LV remodeling.

**Study Type:**

Animal model, longitudinal.

**Animal Model:**

Eighteen lambs (6 months, male, n = 14; female, n = 4; 25.2 ± 4.5 kg).

**Field Strength/Sequence:**

Two‐dimensional balanced steady‐state free precession (bSSFP) and 3D inversion recovery fast low angle shot (IR‐FLASH) sequences at 3 T.

**Assessment:**

Seven lambs underwent test–retest imaging to assess FT interstudy reproducibility. MI was induced in the remaining 11 by coronary ligation with MRI being undertaken before and 15 days post‐MI. Injury size was measured by late gadolinium enhancement (LGE) and LV volumes, LV mass, ejection fraction (LVEF), and wall thickness (LVWT) were measured, with FT measures of global and segmental radial, circumferential, and longitudinal strain.

**Statistical Tests:**

Sampling variability, inter‐study, intra and interobserver reproducibility were assessed using Pearson's correlation, Bland–Altman analyses, and intra‐class correlation coefficients (ICC). Diagnostic performance of segmental strain to predict LGE was assessed using receiver operating characteristic curve analysis. Significant differences were considered *P* < 0.05.

**Results:**

Inter‐study reproducibility of FT was overall good to excellent, with global strain being more reproducible than segmental strain (ICC = 0.89–0.98 vs. 0.77–0.96). MI (4.0 ± 3.7% LV mass) led to LV remodeling, as evident by significantly increased LV volumes and LV mass, and significantly decreased LVWT in injured regions, while LVEF was preserved (54.9 ± 6.9% vs. 55.6 ± 5.7%; *P* = 0.778). Segmental circumferential strain (CS) correlated most strongly with LGE. Basal and mid‐ CS increased significantly, while apical CS significantly decreased post‐MI.

**Data Conclusion:**

FT is reproducible and compensation by hyperkinetic remote myocardium may manifest as overall preserved global LV function.

**Evidence Level:**

N/A

**Technical Efficacy:**

Stage 2

In the setting of myocardial infarction (MI), cardiac MRI is widely regarded as the noninvasive reference standard imaging technique for the measurement of left ventricular (LV) volumes, global function, and reversible and irreversible myocardial injury by late gadolinium enhancement (LGE).^1^, ^2^ However, conventional ventricular volumetry quantifies gross systolic function with limited information about region specific myocardial dysfunction after MI. Moreover, conventional 2D LGE may lack sensitivity in detecting small or heterogeneous areas of MI, potentially leading to partial volume effects and an overestimation of MI size.^2^ Feature tracking (FT) and 3D LGE imaging may overcome these limitations, allowing the characterization of segmental myocardial strain and more precise localization and extent assessment of an MI.

Since its introduction in 2009, FT has been widely accepted and validated in clinical settings.^3^ It has excellent reproducibility in humans^4^, ^5^ and has been validated against myocardial tagging.^6^, ^7^ FT quantifies segmental and global myocardial strain by tracking the movement of distinct patterns, or “features,” in a cardiac cine loop by employing an optical flow technique.^5^, ^8^ Strain analysis by FT can be used to quantify segmental LV myocardial function without dependence on operator experience, unlike visual analysis, which is heavily dependent on operator experience.^9^ Moreover, FT can be applied on conventionally acquired cardiac cine images and does not require a secondary sequence as in myocardial tagging.^3^ We have previously reported visual confirmation of LV regional dysfunction manifesting as segmental hypokinesia or akinesia by cardiac MRI following an MI by coronary ligation in sheep.^10^, ^11^ 2D LGE imaging was also employed to visualize the MI. However, MI size and its relationship with regional myocardial dysfunction were not specifically investigated.

The utility of FT has been explored in other preclinical models, but its use in a sheep model of MI is unclear.^12^, ^13^, ^14^ 3D LGE imaging provides enhanced image quality and may overcome some of the limitations of using 2D LGE in a sheep MI model.^10^, ^11^, ^15^ The hypothesis of this study was that a more comprehensive method of characterizing LV myocardial injury and associated myocardial dysfunction might be a sophisticated approach to studying LV remodeling following MI, and may provide a sensitive methodology for future investigation of novel therapies for MI. Thus, the aim of this study was to validate both FT and 3D LGE in sheep, comparing the findings against post‐mortem histological findings.

## Methods

### Ethical Considerations

All experimental protocols were reviewed and approved by the Animal Ethics Committee of the South Australian Health and Medical Research Institute and abide by the Australian Code of Practice for the Care and Use of Animals for Scientific Purposes developed by the National Health and Medical Research Council.^16^ Merino lambs (~6 months old) were housed in an indoor facility with a constant ambient temperature of 20–22°C and a 12‐hour light/dark cycle. Lambs were housed in individual pens in view of other sheep and had *ad libitum* access to food and water. All investigators understood the ethical principles outlined in Grundy et al.^17^

### Baseline MRI

Merino sheep (n = 18; male, n = 14; female, n = 4; weight 25.2 ± 4.5 kg) underwent baseline MRI using a 3.0 Tesla scanner (Skyra, Siemens Healthineers, Erlangen, Germany). Animals were fasted for 12 hours prior to induction with diazepam (0.3 mg/kg) and ketamine (5 mg/kg). Following intubation, anesthesia was maintained with 2%–3% isoflurane (Lyppards, South Australia, Australia). The animals were scanned in a left lateral decubitus position with a multichannel phased array body coil positioned over the chest. Thoracic localizers were collected in axial, sagittal, and coronal orientations using a balanced steady state free precession (bSSFP) sequence. Cardiac position and axis were determined using the localizers, allowing for prescription of cine bSSFP acquisitions in two‐chamber (2CH), three‐chamber (3CH), and four‐chamber (4CH) orientations. The 2CH and 4CH long‐axis views were then used to prescribe a stack of short‐axis cine slices through the ventricular mass using the following scan parameters: flip angle = ~50°; temporal resolution = ~30 msec; number of views per segments = 9; echo time (TE) = 1.38 msec; repetition time (TR) = 3.2 msec; field of view (FOV) = 300 mm; phase oversampling = 50%; slice‐thickness = 6.0 mm; distance factor = 10%; in‐plane resolution = 1.44 mm × 1.44 mm; number of slices = 15–19; number of signal averages (NSA) = 3; typical acquisition time = ~8 minutes. The temporal resolution used in the sequence yielded >15 true cardiac phases (range 20–25 phases). During 3 T imaging, frequency scouts were performed, and appropriate frequency shifts were applied to prevent artifacts (black‐bands) degrading the LV visual. An arterial blood pressure waveform was processed in LabChart7 (ADInstruments) to generate a cardiac gating signal using the interval between peaks, corresponding to the length of cardiac cycle, as previously described.^11^

The imaging protocol was repeated in seven subjects during the same scanning session for test–retest analysis. In one subject, the test–retest was performed on two separate days (baseline and 15 days post‐MI), resulting in a total of sixteen datasets. Following the completion of the initial scan session, the study was concluded, and the same subject was subsequently registered as a new patient for the retest study. The imaging protocol was repeated for the retest study, with newly acquired localizers. No medication was administered to the subjects between the initial and retest scans.

### Cardiac Surgery

A subset of lambs (n = 11) underwent cardiac surgery while under general anesthesia as for baseline MRI. Following thoracotomy, the heart was exposed, and lignocaine was administered (intravenous slow drip; 100 mg/500 mL) prior to incision of the pericardium. The second diagonal branch of the left anterior descending (LAD) coronary artery was ligated by placing a silk suture around the vessel. Ischemia was evident as blanching of the LV myocardium.^10^, ^18^ The thoracotomy incision was then sutured closed, and antibiotics administered (intramuscular; 3.5 ml of Duplocillin and 2 mL dihydrostreptomycin in sterile saline; Lyppard, Australia) daily for 3‐day post‐surgery. Meloxicam (0.5 mg/kg, subcutaneously; Troylab) was given as analgesia the day before and day of surgery.

### Follow‐Up Cardiac MRI

A follow up cardiac MRI study was performed at 15 days (d) post‐MI surgery using the same protocol as described in baseline MRI to evaluate the progression of myocardial injury and assess cardiac function and remodeling. Gadolinium (Gd‐DTPA; 0.1 mmol/kg; intravenous; jugular vein) was administered and a 3D inversion recovery fast low angle shot (IR‐FLASH) sequence with respiratory gating was employed for LGE imaging.^15^ The following scan parameters were used for 3D IR‐FLASH: voxel size = 1.2 × 1.2 × 1.8 mm (interpolated to 0.6 × 0.6 × 0.9 mm); number of slices = ~80; TR = 597 msec; TE = 1.27 msec; TI = 450 msec; slice resolution = 50%; segments = 45; scan time = ~8 minutes. A respiratory gating navigator was placed across the diaphragm.

### Cardiac MRI Post‐Processing

All cardiac MRI acquisitions were assessed by SKSC (6 years of experience), supported by MS (22 years of experience), for exclusion from the study dataset due to flow, black‐band artifacts, and inadequate image prescriptions.

#### VENTRICULAR VOLUMETRY

The LV was segmented using semiautomatic endo‐ and epicardial contours to measure end‐diastolic volume (EDV), end‐systolic volume (ESV), stroke volume (SV), and ejection fraction (EF). LV myocardial mass was measured in systole.^19^ LV wall thickness (WT) was quantified at segmental levels in systole to assess ventricular remodeling. Post‐processing was performed by SKSC supported by MS, using CVI42 version 5.13.5 (Circle Cardiovascular Imaging, Canada).

#### FT MYOCARDIAL STRAIN

FT analysis was performed using a feature‐tracking algorithm (CVI42). The end‐diastolic contours were automatically propagated throughout the cardiac cycle using the FT algorithm. Contours were confirmed by visual inspection. In the case of inadequate tracking, the contours were adjusted, and the analysis repeated. Top basal short‐axis slices were excluded from FT analysis by mitral annular plane systolic excursion (MAPSE) due to the longitudinal through‐plane motion of the LV in systole.^20^ Similarly, the end‐apical slice was also excluded to account for through‐plane motion. 2D global radial strain (GRS) and global circumferential strain (GCS) were measured from the short‐axis cine stack. Global longitudinal strain (GLS) was calculated as the mean of the longitudinal strain measured in each of the three long‐axis planes (2CH, 3CH, 4CH). Long‐axis extents (LAX) were placed on all acquired long‐axis views to define the longitudinal range of the LV to avoid under sampling of strain due to the elongated apical LV anatomy present in sheep. The American Heart Association (AHA) 16‐segment model was used to quantify and present segmental myocardial strain measurements.^21^ Segments with inadequate tracking were excluded. Basal, mid, and apical strain was calculated as the mean of the respective segments (segments: 1–6 = basal; 7–12 = mid; 13–16 = apical). Strain analysis was repeated three times to assess sampling variability and intraobserver reproducibility in all datasets (SKSC). Interobserver reproducibility was assessed in a subset (n = 13) of the dataset (SKSC and AR, 3 years of experience). Mechanical dispersion was assessed qualitatively in each subject by noting asynchronous circumferential strain in all slices over the cardiac cycle.

#### QUANTIFICATION OF MI BY 3D IR‐FLASH

**FIGURE 1:**
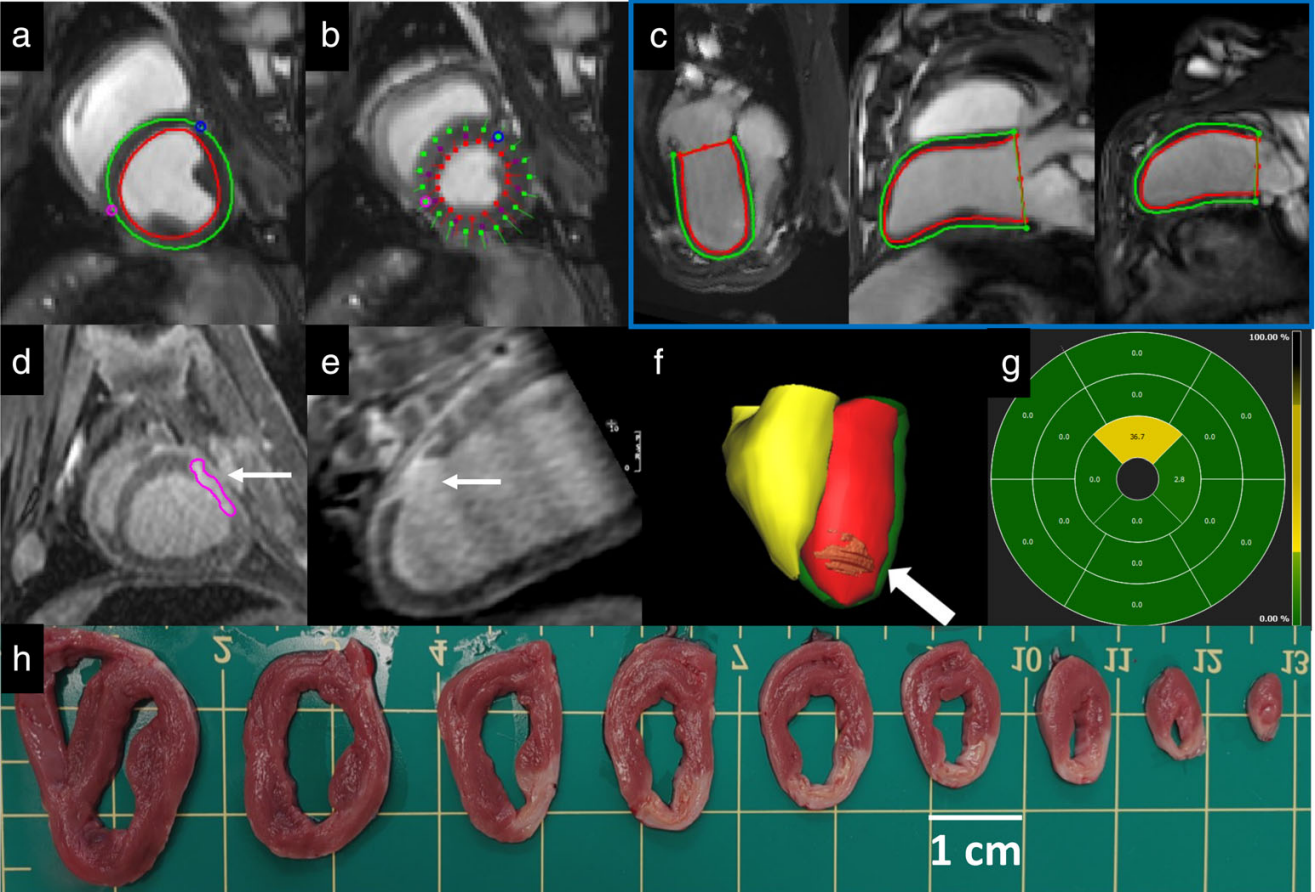
Post‐processing of MRI data and tissue processing. Endocardial (red) and epicardial (green) contours on the left ventricle in diastole (**a**) and feature tracking in systole (**b**). Contours applied in three‐apical long‐axis views; 4‐, 3‐, 2‐chamber views, respectively (**c**). Quantification of late gadolinium enhancement (white arrow) on 3D IR‐FLASH acquisition in the short‐axis (**d**) and 2‐chamber view (**e**). 3D model of the left ventricle (red) and LGE overlay (**f**; white arrow). Bullseye plot of LGE on a 16‐segment model (yellow; g). Short‐axis slices (~1 cm thick) of stained sheep heart acquired at post‐mortem showing blanching of nonviable tissue (**h**).

Nonviable scar resulting from the MI was assessed at 15d by manual segmentation of LGE on the 3D IR‐FLASH acquisition (Fig. 1). The total mass of MI was measured and expressed as the percentage of LV mass. The regional size and extent of myocardial injury were quantified and reported as relative % LGE using the AHA model (Fig. 1a–g). Segmentations were performed by SKSC supported by MS.

### Post‐Mortem Tissue Collection

Sixteen days after the MI surgery, sheep were humanely killed with an over dose of sodium pentobarbitone (20 mL; Virbac, Peakhurst, New South Wales, Australia). The heart was dissected, and then sliced into sections ~1 cm thick. Each section was stained with 2,3,5‐triphenyltetrazolium chloride (TTC; Fig. 1h).^10^

### Statistical Analysis

Intraclass correlation (ICC) and coefficient of variation (COV) were calculated to compare the FT sampling variability of single measures and the average of three measurements.^5^, ^22^ Test–retest analysis was performed by comparing strain measurements from the two sequential scans by linear regression, Pearson's correlation, and Bland–Altman plots. Segmental strain measures were compared using ICC analysis. The intraobserver and interobserver variability of FT strain measurements were assessed using the same set of statistical tests. The diagnostic performance of segmental CS, RS, and LS in detecting myocardial injury by LGE was assessed using receiver operating characteristic curve (ROC) analysis. ROC analysis was repeated by categorizing infarct sizes into subgroups based on thresholds of LGE at 10% intervals to classify segmental injury (i.e., 0%, 10%, … 40%). For example, with the 0% threshold, all segments with LGE >0% were deemed injured and segments greater than or equal to 10%, 20%, 30%, or 40% LGE were classified as injured. Bootstrapping was performed by subject (n = 200 iterations) to estimate AUC and 95% CI for each respective ROC analysis. The diagnostic performance of segmental strains in detecting LGE >0% was compared using the Kruskal–Wallis rank sum test, followed by post hoc analysis using Dunn's test. The segmental strain exhibiting the highest area under the curve (AUC) was selected and the percentage change of segmental strain was calculated using paired measurements from baseline and 15d post‐MI MRI studies. Segmental and global strains between baseline and 15d post‐MI were compared using paired *t*‐tests. All data analyses were performed with Graphpad (Prism 8.0, San Diego, USA) and R (V4.1.2, Vienna, Austria). ICC estimates and 95% CI were calculated using the irr package in R (V0.84.1), based on a mean‐rating, absolute‐agreement, two way mixed‐effect model. In our analysis, ICC values <0.5 indicate poor reliability, values between 0.5 and 0.75 indicate moderate reliability, values between 0.75 and 0.9 indicate good reliability, and >0.9 indicate excellent reliability.^23^ ROC analysis was performed using the ROCit package in R (V2.1.1). For segmental strain analysis, a compound symmetric correlation matrix was assumed.^24^ The analysis was performed using the nlme package in R. *P* < 0.05 was considered statistically significant.

## Results

### MR Image Quality

All short‐axis cine acquisitions (n = 38) acquired from each of the animals (n = 18) exhibited suitable image quality for LV ventricular volumetry and FT. Some long‐axis cine acquisitions were either not acquired (n = 7) or excluded due to flow artifacts, black‐band artifacts, and difficulties with image prescriptions (4CH: 0/38, 3CH: 5/38, and 2CH: 2/38). For test‐retest analysis, all short‐axis and 4CH cine acquisitions (n = 16) were suitable, but some 3CH (3/16) and 2CH (4/16) images were excluded due to flow artifacts, prescription issues, or were not collected.

### Sampling Method and Variability of FT

The comparison between the averaged global strain measures derived from three measurement repetitions and one measurement repetition showed no improvement in sampling variability (GRS, ICC = 0.994 [0.983–0.998]; GCS, ICC = 0.993 [0.98–0.998]; GLS, ICC = 0.988 [0.961–0.996]) (Table S1 in the Supplemental Material). There was excellent agreement with low bias between short‐axis derived global strain measurements when comparing the average of three repetitions and one repetition measurements. Segmental RS (bias = −0.09 ± 2.75; ICC = 0.977 [0.970–0.982]) and CS (bias = −0.004 ± 0.72; ICC = 0.984 [0.979–0.987]) demonstrated excellent agreement with near‐zero bias. GLS measurements had excellent agreement with low bias (bias = −0.11 ± 0.40; ICC = 0.988 [0.961–0.996]), although segmental LS had good to moderate reliability and slight bias (bias = 0.70 ± 4.10; ICC = 0.766 [0.693–0.824]). Given this finding, single repeat measurements were used for all other data presented below.

### FT Interstudy Reproducibility

**FIGURE 2:**
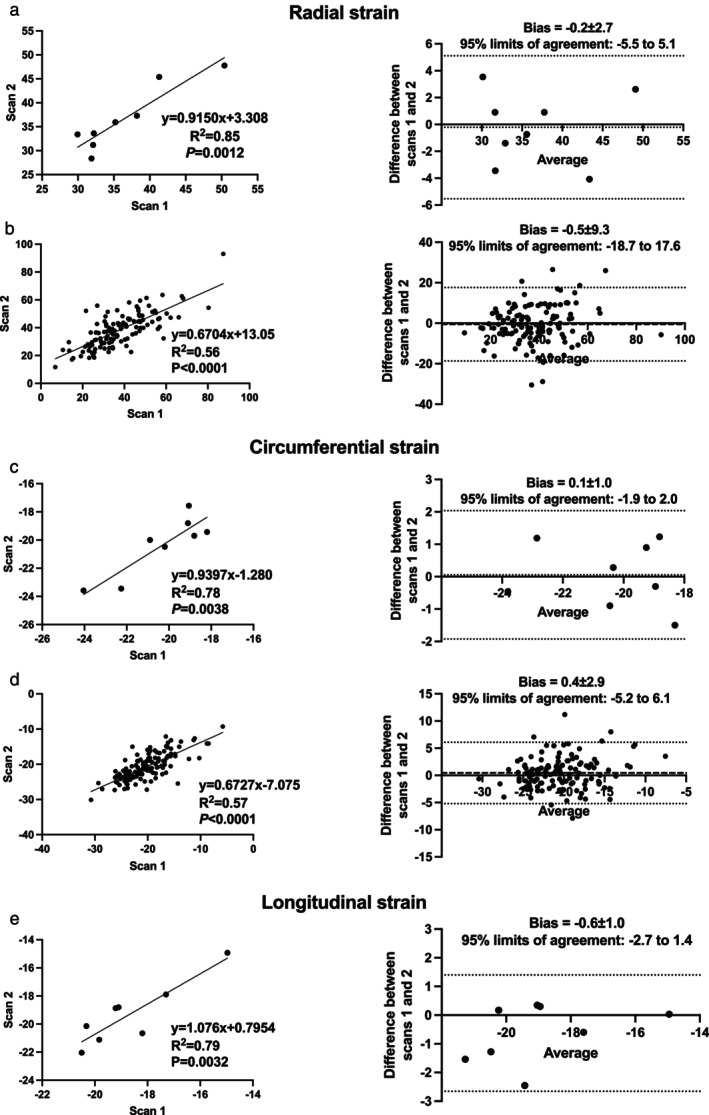
Test–retest analysis of global and segmental strain. Linear regression and Bland–Altman analyses comparing global and segmental radial strain (**a‐b**), circumferential strain (**c‐d**), and global longitudinal strain (**e**).

The heart rate was comparable between scans (85 ± 11 vs. 84 ± 9 bpm; *P* = 0.6822). Test–retest values of GRS (*R* ^2^ = 0.85; bias = −0.2 ± 2.7; 95% limits of agreement: −5.5 to 5.1) and GCS (*R* ^2^ = 0.78; bias = 0.1 ± 1.0; 95% limits of agreement: −1.9 to 2.0) demonstrated a high degree of correlation and no significant bias (Fig. 2). Segmental RS (*R* ^2^ = 0.56; bias = −0.5 ± 9.3; 95% limits of agreement: −18.7 to 17.6) and CS (*R* ^2^ = 0.57; bias = 0.4 ± 2.9; 95% limits of agreement: −5.2 to 6.1) also demonstrated good correlation and agreement with a small bias. Segmental RS (ICC = 0.75 [0.67–0.80]) and CS (ICC = 0.75 [0.68–0.81]) showed good to moderate interstudy reliability. There was a high degree of correlation between GLS measures from the sequential scans without significant bias (Fig. 2e). There was a significant but modest correlation between 4CH‐GLS measured from the sequential scans (*y* = 0.792*x* − 5.189; *R* ^2^ = 0.60), and good agreement with a small bias (bias = 1.3 ± 2.0; 95% limits of agreement: −2.65 to 5.21). There was insufficient data to compare 3CH and 2CH LS from the sequential scans.

### Intra and Interobserver Reproducibility of FT

Intraobserver reproducibility was overall excellent (Table S2 in the Supplemental Material). Global and segmental strains generally had excellent correlation, excellent reliability, and nonsignificant bias. Interobserver reproducibility was lower compared to intraobserver reproducibility. Segmental strain reproducibility was lower than global strain reproducibility.

### Diagnostic Performance of Segmental Strain for Myocardial Injury

**FIGURE 3:**
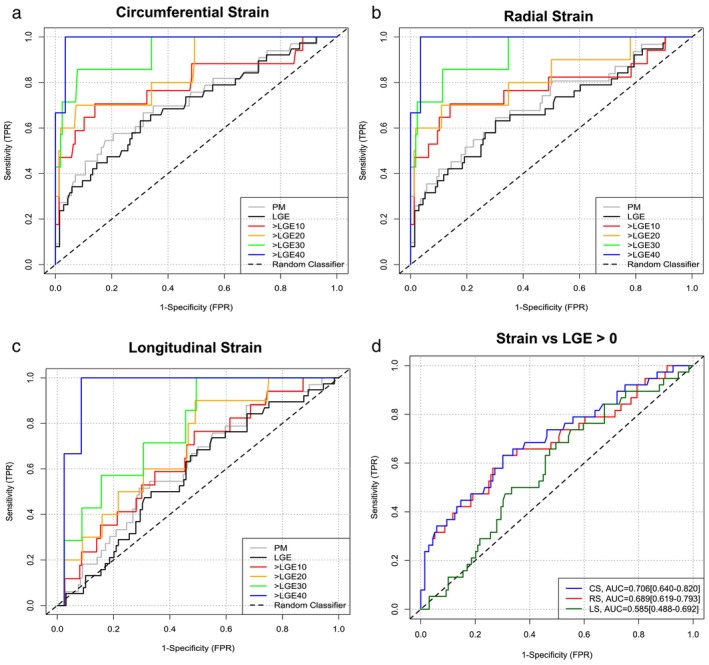
Receiver operating characteristic analysis of diagnostic performance of segmental strains to classify myocardial injury by late gadolinium enhancement (LGE). In all segmental strain, the diagnostic performance (area under the curve [AUC]) increased with increasing LGE thresholds used for injury classification (**a‐c**). Using segments with LGE >0, circumferential strain demonstrated the highest diagnostic performances, while longitudinal strain demonstrated lowest diagnostic performance (**d**). PM = post‐mortem.

There were 38 segments with LGE >0, 17 segments with LGE ≥10%, 10 segments with ≥20%, 7 segments with ≥30%, and 3 segments with ≥40%. Classification of injury by histological confirmation at post‐mortem (Fig. 3) and by LGE values greater than 0 resulted in the lowest diagnostic accuracies in all segmental strains (CS, ICC = 0.706 [0.647–0.799]; RS, ICC = 0.689 [0.619–0.793]; LS, ICC = 0.585 [0.488–0.692]; Table 1). The diagnostic performance of segmental strain increased with increasing LGE threshold used to classify injury. LGE threshold of ≥40% resulted in the highest diagnostic accuracy of all strains (CS, ICC = 0.991 [0.884–1.000]; RS, ICC = 0.991 [0.860–1.000]; LS, ICC = 0.957 [0.647–0.992]). There was a difference between the segmental strains in their diagnostic performances in classifying LGE (*P* < 0.001); AUCs of CS (*P* < 0.001) and RS were higher than LS (*P* < 0.001), while CS and RS were comparable (*P* = 0.1178) (Fig. 3d).

**TABLE 1:** Optimal Cut‐Off Values for Segmental Strains and AUC at Different LGE Thresholds for Injury Classification

	PM	LGE	LGE ≥10%	LGE ≥20%	LGE ≥30%	LGE ≥40%
Circumferential strain						
Cut‐off (%)	−16.02	−17.77	−14.87	−12.96	−12.96	−11.78
Sensitivity	0.548	0.632	0.706	0.7	0.857	1
Specificity	0.799	0.699	0.86	0.927	0.922	0.965
AUC [95% CI]	0.724 [0.640–0.820]	0.706 [0.647–0.799]	0.799 [0.712–0.954]	0.864 [0.745–0.973]	0.942 [0.843–0.996]	0.991 [0.884–1.000]
Radial strain						
Cut‐off (%)	28.3	28.3	21.81	19.59	19.59	16.67
Sensitivity	0.645	0.632	0.706	0.7	0.857	1
Specificity	0.698	0.699	0.860	0.890	0.886	0.965
AUC [95% CI]	0.718 [0.626–0.814]	0.689 [0.619–0.793]	0.784 [0.671–0.951]	0.834 [0.687–0.973]	0.934 [0.832–0.996]	0.991 [0.860–1.000]
Longitudinal strain						
Cut‐off (%)	−13.68	−16.78	−16.3	−16.3	−16.3	−8.52
Sensitivity	0.515	0.684	0.765	0.9	1	1
Specificity	0.709	0.504	0.513	0.51	0.506	0.915
AUC [95% CI]	0.545 [0.481–0.608]	0.585 [0.488–0.692]	0.652 [0.543–0.759]	0.706 [0.545–0.829]	0.777 [0.587–0.902]	0.957 [0.647–0.992]

There were 38 segments with LGE >0, 17 segments with LGE ≥10%, 10 segments with LGE ≥20%, 7 segments with LGE ≥30%, and 3 segments with LGE ≥40%. AUC = area under the curve; PM = post‐mortem; LGE = late gadolinium enhancement.

### LV Dysfunction and Remodeling Following Myocardial Infarction

#### MYOCARDIAL INJURY AND CONVENTIONAL MRI LV PARAMETERS

**FIGURE 4:**
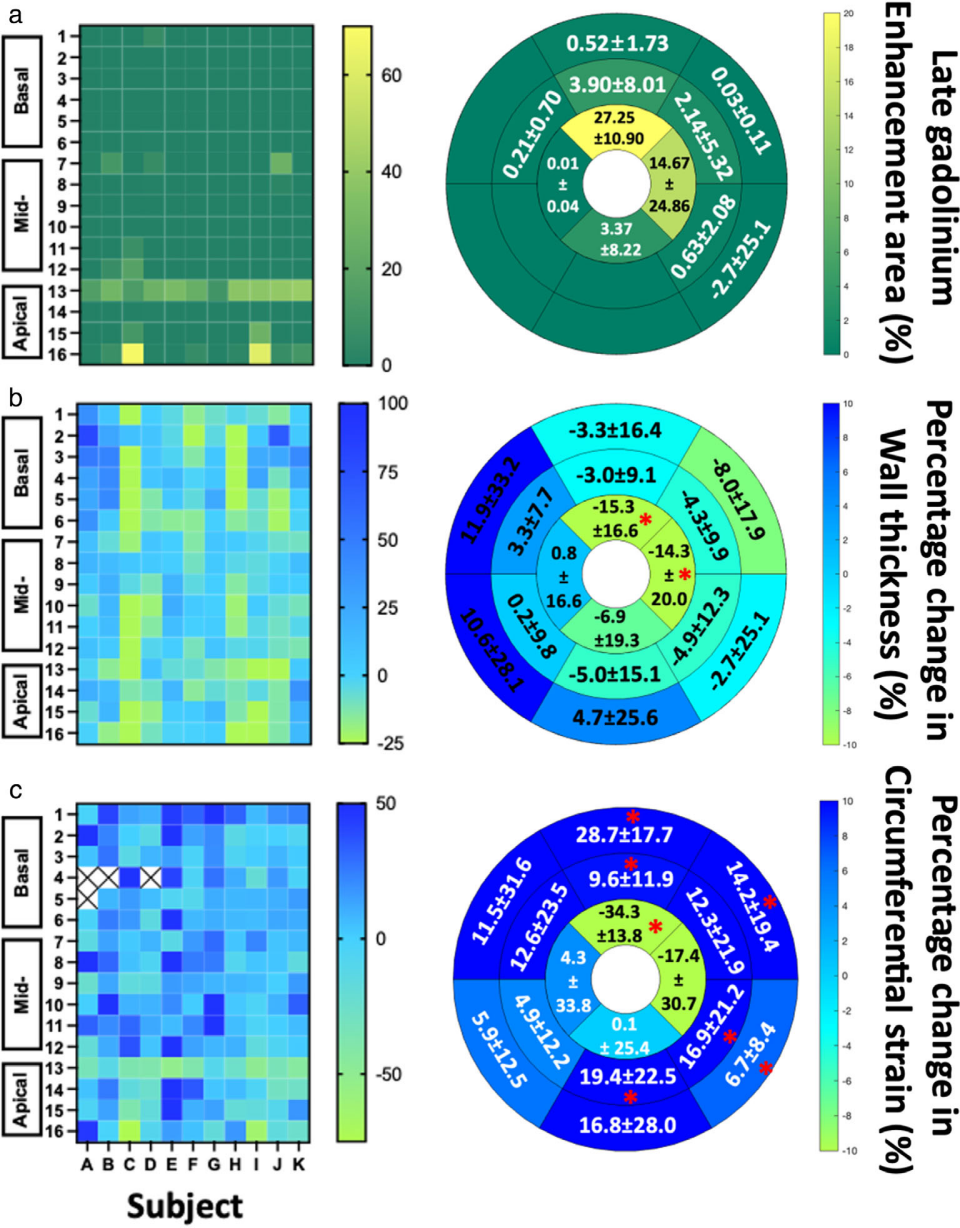
Heat maps of the outcome of each subject in the study and bullseye plot of collated data for the respective variable. Late gadolinium enhancement data for each subject (letters A–K; x‐axis) in each segment (1–16 on the y‐axis) and the bullseye plot of the mean and SD of each segment (**a**). Percentage change in wall thickness (**b**) and circumferential strain (**c**). *P < 0.05; paired Student's *t*‐test, n = 11.

3D LGE imaging provided excellent image quality and allowed clear delineation and quantification of myocardial injury (Figs. 1 and 4; Video S1). The size of nonviable tissue 15d post‐MI was 1.86 ± 1.78 g (range: 0.25–6.1 g) and MI size as % of LV mass was 4.0 ± 3.7%. All injuries (n = 11) transitioned from partially subendocardial to fully transmural from mid to apex (Video S1). Our method of MI by coronary ligation resulted in injury in the apical anterolateral LV (Fig. 4a). There was LV dilatation following MI (Video S2) as evidenced by statistically significant increases in LVEDV, LVESV, and SV. The cardiac output increased significantly with comparable heart rates between baseline and follow‐up (*P* = 0.420). Overall, LVEF was comparable between baseline and follow‐up (*P* = 0.778; Table 2). There was a significant increase in LV mass from baseline to follow‐up. LVWT were significantly lower in the apical anterior (7.7 ± 1.5 vs. 6.4 ± 1.1) and lateral segments (6.4 ± 1.4 vs. 5.3 ± 0.9) at 15d post‐MI compared with baseline (Video S3). There was no change in LVWT in apical septal (6.7 ± 1.3 vs. 6.6 ± 0.8; *P* = 0.756) and inferior segments (5.4 ± 1.2 vs. 4.9 ± 0.9; *P* = 0.202). There was a negative percentage change in LVWT in basal and mid‐anterolateral segments at 15d post‐MI compared with baseline, but without statistical changes (Fig. 4).

**TABLE 2:** Paired LV Volume and FT Measurements at Baseline and 15d Post‐MI

	Baseline	15d	*P*
LV end diastolic volume	66.3 ± 14.6	79.0 ± 16.2	**0.001**
LV end systolic volume	29.9 ± 8.8	35.3 ± 9.1	**0.051**
LV stroke volume	36.4 ± 8.2	43.8 ± 9.7	**<0.001**
Heart rate	84.6 ± 12.0	82.8 ± 13.0	0.420
LV ejection fraction	54.9 ± 6.9	55.6 ± 5.7	0.778
Cardiac output (L/min)	3.0 ± 0.7	3.6 ± 0.6	**0.002**
Cardiac index (L/min/kg)	0.14 ± 0.04	0.15 ± 0.02	**0.039**
LV mass (g)	43.5 ± 7.6	46.7 ± 5.9	**0.005**
Feature tracking			
Global radial strain	29.8 ± 5.2	32.7 ± 6.8	0.131
Global circumferential strain	−17.8 ± 1.8	−18.6 ± 2.0	**0.196**
Global longitudinal strain	−17.2 ± 2.8	−14.6 ± 2.2	**0.011**
4‐chamber longitudinal strain	−18.2 ± 2.3	−16.5 ± 2.6	**0.041**
3‐chamber longitudinal strain	−17.3 ± 4.1	−14.0 ± 2.5	**0.015**
2‐chamber longitudinal strain	−16.0 ± 3.4	−13.2 ± 2.5	**0.034**

Values represent mean ± SD, n = 11. Bold indicates *P* < 0.05.

#### REGIONAL CHANGES IN MYOCARDIAL STRAIN AND CORRELATION WITH LGE

**FIGURE 5:**
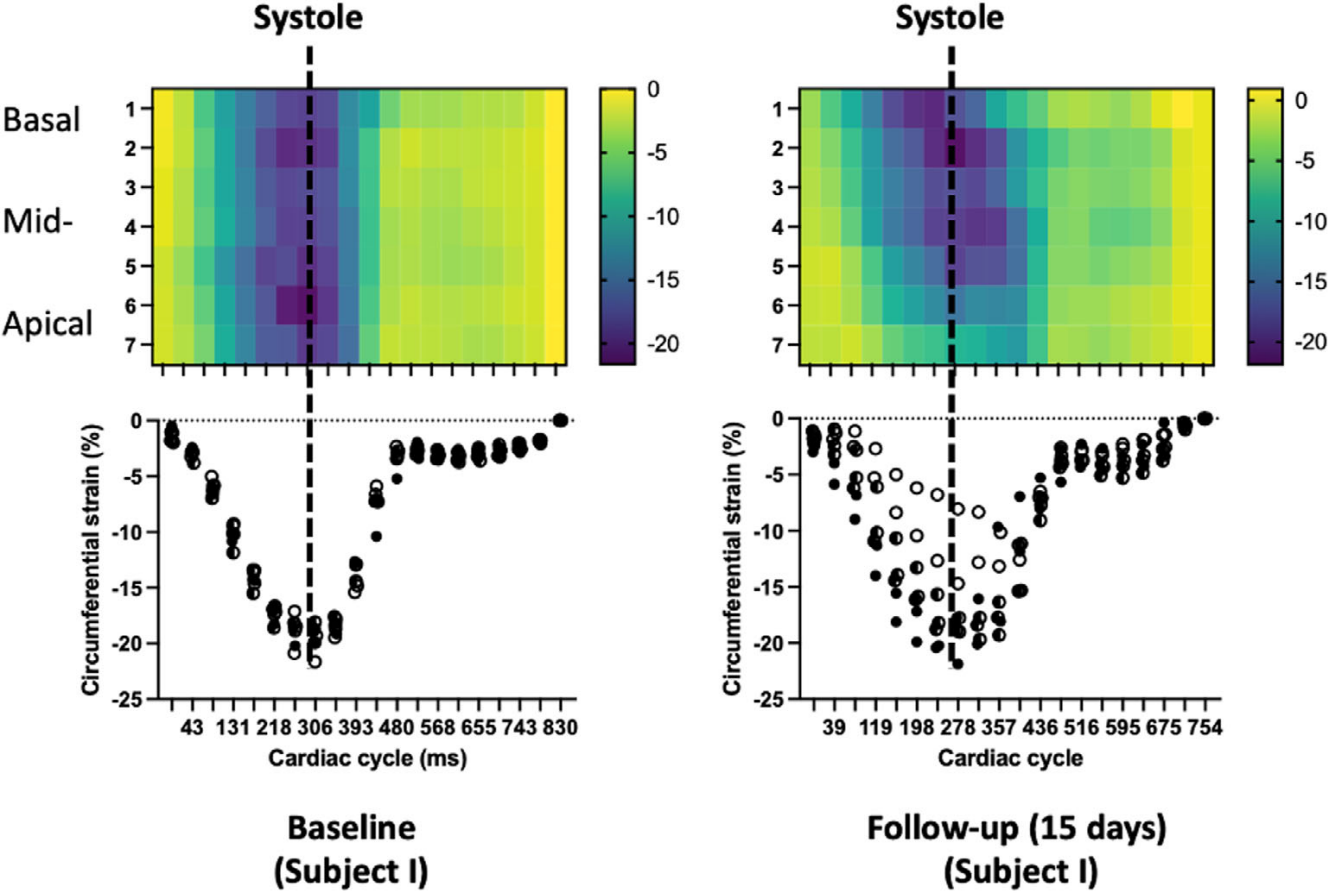
Mechanical dispersion of LV (subject I). The circumferential strain in all short‐axis slices of the LV (y‐axis) over time (cardiac cycle; x‐axis) plotted on both a heat map (top) and graph (bottom), at baseline (left) and 15 days follow‐up (right). The strain values are less centered around the systolic time point at follow‐up, and apical strain has relatively low peak strain and its incidence is delayed by ~80 msec when compared with the other slices.

Given that segmental CS demonstrated the best diagnostic performance, both RS and LS at segmental levels were not included in correlation analyses. Basal LVCS increased significantly from baseline to 15d post‐MI (−17.6 ± 1.8 vs. −19.7 ± 2.0) as well as mid‐LVCS (−17.5 ± 1.7 vs. −19.3 ± 1.7), while apical LVCS significantly decreased (−18.6 ± 3.1 vs. −16.0 ± 3.3). GLS and LS in all long‐axis planes significantly decreased at follow‐up compared with baseline (Table 2). Paired *t*‐tests comparing segmental CS measures between baseline and 15d post‐MI revealed both increases and decreases in segmental CS in different parts of the LV (Fig. 4). There were decreases in the percentage change of segmental CS in segments with LGE, in the anterior and lateral apical LV (Video S4; Fig. 4). Paired *t*‐test revealed statistically lower apical anterior LVCS at 15d post‐MI compared with baseline (−20.3 ± 2.1 vs. −13.3 ± 3.4; *P* < 0.001), but not in the apical lateral LVCS (−17.9 ± 2.9 vs. −14.1 ± 4.8; *P* = 0.100). Prominent myocardial wall thinning coincided with the same two apical segments with diminished segmental CS (Fig. 4). Moreover, these regions of diminished percentage change of segmental CS coincided with segments of injury confirmed by LGE (Fig. 4). In select remote areas of LV myocardium, the segmental CS increased, while the percentage change in CS increased in all remote myocardium from baseline to 15d post‐MI (Fig. 4). Mechanical dispersion was observed in 8/11 subjects at follow‐up (Fig. 5; Fig. S1 in the Supplemental Material).

## Discussion

In this study, FT and 3D LGE were applied in a sheep model of MI. Overall, the data suggest that FT is highly repeatable and sensitive for detecting myocardial injury resulting from coronary ligation induced MI. Moreover, the data suggest that LV remodeling occurs by 15d post‐MI, evident as ventricular dilatation, increased myocardial mass, and thinning of myocardium in regions of ischemic injury. Surprisingly, there was no effect of MI on global LV function parameters including LVEF, GRS, and GCS. However, notably diminished segmental strain was detected in regions of myocardial injury while viable, remote myocardium demonstrated compensatory increases in strain that may explain the preserved LV global function.

Measurement repetitions are generally employed in FT studies to improve reproducibility.^5^, ^12^, ^13^, ^22^ Although past animal FT studies using measurement repetitions report good to excellent reproducibility, comparison with single measures was not provided.^12^, ^13^ Clinical FT studies generally report improved reproducibility and decreased variability from three repeated measurements compared with a single measurement, but this varies by study.^5^, ^22^ The effect of repeated measures was most pronounced for GRS and/or LV torsion when comparing different vendors.^5^, ^22^ At intravendor levels, the benefit of repeated measurements has been reported to be relatively low in GRS and GCS, and both have demonstrated excellent reproducibility using CVI42.^5^ A study of multiple vendors that did not include Circle software found improvement with repeated measurements of GRS and RV GLS.^22^ However, comparison with past studies may not be appropriate as study design varies widely, including image acquisition protocol, observer experience, and inconsistent use of FT analysis software. When considering the use of measurement repetitions, careful consideration of time vs. cost effectiveness is warranted, as repetitions can increase analysis time up to 3‐fold.^5^ Our data suggest a high degree of reliability and reproducibility between the two sampling methods, justifying the use of single measurements for the majority of parameters, while the use of segmental LS requires further investigation.

Interstudy reproducibility was overall excellent, and the higher reproducibility of global strain measures compared with segmental measures is in keeping with the literature; global strain is widely accepted as more robust and reproducible than segmental strain.^4^, ^7^, ^25^, ^26^ This was the case with both intra and interobserver reproducibility, with generally lower segmental strain reproducibility compared with global strain. Although RS and CS demonstrated similar reproducibility in our study, CS is more reliable in sheep with consistently lower COVs compared with RS, which is in keeping with an emerging consensus that CS is more robust than RS.^4^, ^5^, ^13^, ^27^

The reproducibility of FT in the current study was more favorable than prior clinical reproducibility studies.^4^, ^5^, ^9^, ^25^ One explanation may be the reduced complexity of LV structure in sheep than human hearts. The sheep heart has larger papillary muscles with non‐visible trabeculae, whereas in human hearts the papillary muscles are relatively small and the LV has extensive trabeculae.^28^ As FT measurements depend on the tracked features based on the contours placed on the LV, the less complex LV structure of the sheep may have contributed to the lower sampling variability seen in our study. Moreover, the simpler LV sheep structures likely helped to reduce false tracking due to through‐plane motion compared with the human heart.

The lower reproducibility of LS reported in this study may be attributed to the geometry of the sheep heart, which has an elongated apex bending anteriorly toward the chest wall and does not follow the typical prolate ellipsoid geometry found in humans.^29^ Thus, acquiring true apical 4CH and 2CH long‐axis views is not possible in sheep and therefore foreshortening of the apical LV in long‐axis acquisitions is unavoidable. Due to this lack of an important landmark for obtaining true long axis views of the LV, there may be variability in the long‐axis prescriptions in our study, ultimately leading to variability in segmental LS. However, this effect may be mitigated in the assessment of GLS and LS in the respective long‐axis views as these are the mean values of several LS segments, a finding in keeping with a previous study.^26^

In this study, the increasing diagnostic performance of segmental strain with increasing LGE threshold cut‐off for myocardial injury demonstrates the relationship between myocardial function and extent of injury.^30^ Moreover, higher diagnostic performance of short‐axis derived segmental strain to predict LGE is in keeping with previous studies.^30^, ^31^ The finding of relatively less reliable segmental LS is in agreement with past studies and also may be explained by the reliance on some degree of interpolation between the long‐axis acquisitions as part of the FT analysis algorithm. Although the highest diagnostic performance was noted using cut‐offs of LGE ≥40%, this finding was based on less than 2% of segments with LGE ≥40% and therefore warrants careful interpretation.

At follow‐up, all 11 subjects presented with myocardial injuries that transitioned from being subendocardial in mid‐myocardium to fully transmural near the apex. This injury phenotype may be characteristic of the methodology by which MI was induced leading to LV remodeling by 15 days. LV dilatation observed at 15 days post‐MI in this study reproduces findings in past swine MI studies, reporting increased LV volumes at 16d and 30d post‐MI.^32^, ^33^ This suggests that LV dilatation may be a relatively rapid process, occurring in the first 2 weeks following MI, and may occur as early as 3d post‐MI as previously reported in our past sheep MI study.^10^ In addition to LV dilatation, increased LV mass suggest the presence of eccentric hypertrophy and adverse remodeling, which is in keeping with nonviable areas of injury and wall thinning at sites of injury.^34^ There is also evidence of concentric hypertrophy in the remote myocardium, mostly in the septal and inferior LV walls, which may be explained by the classic wall‐stress hypothesis.^35^

Surprisingly, LVEF was preserved, but this finding was accompanied by hyperkinetic remote myocardium as previously described in both clinical and preclinical settings.^32^, ^36^, ^37^, ^38^ The observed compensatory increase in CS is reflective of the contractile reserves of the nonischemic remote myocardium and the hypercontractility in these areas of the myocardium may be explained by the Frank–Starling mechanism and intraventricular unloading from nonischemic to ischemic myocardium.^37^ Of note, the hyperkinesis of remote myocardium and increased stroke work at follow‐up may have been exacerbated by the removal of pericardium during cardiac surgery, as the pericardium exerts influence on the pressure‐volume relationships of the LV.^39^ It is possible that the pericardiotomy influenced the degree of LV remodeling. While LVEF was preserved, GLS and LS in the long‐axis views were reduced, and this may be reflective of impaired shortening of the longitudinal subendocardial fibers, which are more sensitive to ischemia in the early stages of MI.^40^ Moreover, this finding is in keeping with reports of GLS being a powerful independent predictor of mortality in the setting of heart failure with preserved EF as well as exhibiting higher sensitivity to systolic dysfunction than EF.^41^, ^42^

The approach used in this study may be particularly useful in studying subjects presenting with preserved or increased LVEF following an MI, despite notably diminished contractility in injured ischemic segments. Higher resolution imaging of myocardial injury *in vivo* as well as quantification of segmental strain as demonstrated in this study may be employed in future MI preclinical studies, potentially allowing for establishing correlations between regional myocardial function and molecular markers of contractility and cardiomyocyte physiology.

## Limitations

The segmental analyses were performed using a compound symmetric correlation matrix as previously reported by others.^24^ This approach assumes that each segment of the LV is equally dependent to each other and this neglects the spatial correlation between segments, which does not account for interactions between neighboring segments (i.e., tethering effects). The terms hypo‐ and hyperkinesis were used based on qualitative assessment and relative differences in quantitative measures of segmental strain, and warrant further investigation to define thresholds. The effect of pericardiectomy between the two scans could not be controlled for in this study as we ignored the pericardium in attempt to minimize the animals' exposure to anesthesia. Inclusion of a sham surgery protocol or studying the isolated effects of pericardiectomy in sheep may be helpful in future studies involving preclinical models of MI. There was inadequate sample size of selected long‐axis cine images for certain statistical analyses due to inconsistent MRI acquisitions. A standardized cardiac MRI imaging protocol tailored to the sheep heart anatomy may allow for more informative longitudinal imaging in the future. Another potential limitation of our model is the relatively high occurrence of adhesion of LV tissue to the chest wall, which was observed at post‐mortem. The physical adherence of regions of LV injury to the chest wall may have exaggerated the regional wall motion abnormality observed at follow‐up. Diastolic function was not assessed as there was insufficient temporal resolution to assess strain rate, a differential variable. Sheep heart anatomy exhibits notable differences from that of the human heart, which may impact the translatability of these techniques to the clinical setting. The age of the sheep used in this study is younger the typically older human patients with coronary artery disease, and this may lead to differences in the extent of LV remodeling and recovery in the setting of acute MI. The pathophysiology of MI induced by coronary ligation may differ from the commonly atherosclerotic origin seen in the clinical setting, warranting further investigation. However, it should be noted that the size and electrophysiology of the sheep heart is similar to that of the human heart.^43^

## Conclusions

This study presents repeatability data for FT in a sheep model of MI and provides some insight into LV remodeling and the compensatory mechanism of hyperkinesis in remote myocardium manifesting as preserved LVEF. In adjunct with ventricular volumetry by conventional MRI, this study allowed comprehensive characterization of LV physiology and myocardial injury in the setting of MI.

## Author Contributions

Conception or design of the work: JLM, MS, CKM, and JBS. Acquisition or analysis or interpretation of data for the work: SKSC, JLM, and MS. Drafting the work or revising it critically for important intellectual content: SKSC, JLM, and MS. Final approval of the version to be published and agreement to be accountable for all aspects of the work: All.

## Supporting information


**Video S1:** A cine display presenting a sequential stack of 3D late gadolinium images acquired by IR‐FLASH.

**Video S2:** Cine acquisition of an apical short‐axis slice displaying anterolateral regional wall motion abnormality in subject F.

**Video S3:** Two chamber cine views at 15 days post‐myocardial infarction in subjects J (left) and K (right), displaying left ventricular dilatation and anterior wall thinning.

**Video S4:** Apical short‐axis slices of subject F with circumferential strain color overlay, displaying diminished anterolateral strain.

**Data S1:** Supporting Information.

## Data Availability

The data that support the findings of this study are available from the corresponding author, upon reasonable request.
