# Supplementary material for: Post‐Myocardial Infarction Remodeling and Hyperkinetic Remote Myocardium in Sheep Measured by Cardiac MRI Feature Tracking
Source: J Magn Reson Imaging. 2024 Jun 28;61(3):1323–35. doi: 10.1002/jmri.29496 (PMC11803690; doi:10.1002/jmri.29496)
Supplement: Supplementary file 5 — Data S1: Supporting Information. [file JMRI-61-1323-s005.docx]

**Supplemental Tables**

**Table 1**. Comparison of CMR-FT sampling variability derived by single repeat measurements vs averaged measure of three repetitions.

|  | **Mean difference**  **[95% limits of agreement]** | **Standard deviation** | **1 repetition**  **COV** | **3 repetition COV** | **ICC [95% CI]** |
| --- | --- | --- | --- | --- | --- |
| **Global radial strain** | -0.07  [-1.53 – 1.40] | 0.75 | 17.92% | 18.55% | 0.994  [0.983 – 0.998] |
| **Global circumferential strain** | 0.03  [-0.44 – 0.51] | 0.24 | 9.79% | 10.05% | 0.993  [0.980 – 0.998] |
| **Segmental radial strain** | -0.09  [-5.30 – 5.48] | 2.75 | 33.82% | 32.50% | 0.977  [0.970 – 0.982] |
| **Segmental circumferential strain** | -0.004  [-1.42 – 1.42] | 0.72 | 19.80% | 19.13% | 0.984  [0.979 – 0.987] |
| **Segmental longitudinal strain** | 0.70  [-8.75 – 7.34] | 4.10 | 25.91% | 35.58% | 0.766  [0.693 - 0.824] |
| **Global longitudinal strain** | -0.11  [-0.35 – 0.13] | 0.40 | 9.15% | 9.45% | 0.988  [0.961 – 0.996] |
| **4-chamber longitudinal strain** | -0.03  [-1.32 – 1.26] | 0.51 | 15.14% | 14.05% | 0.971  [0.92 – 0.99] |
| **3-chamber longitudinal strain** | 0.14  [-0.98 – 1.25] | 0.57 | 13.53% | 13.71% | 0.977  [0.93 – 0.993] |
| **2-chamber longitudinal strain** | 0.03  [-1.03 – 0.96] | 0.51 | 10.45% | 9.84% | 0.969  [0.896 – 0.991] |

COV, coefficient of variation; ICC, intraclass correlation; CI, confidence interval; data points; n=16 from 8 test-retest scans (8*2)

**Table 2. Intra- and interobserver reproducibility data of CMR-FT strain parameters.**

|  | **Intraobserver** | | | | | | | **Interobserver** | | | | | | |
| --- | --- | --- | --- | --- | --- | --- | --- | --- | --- | --- | --- | --- | --- | --- |
|  | **Linear regression &** | **Bias ± SD** | **95% limits of agreement** | **COV** | **COV 2** | **ICC [95% CI]** | **n** | **Linear regression &** | **Bias ± SD** | **95% limits of agreement** | **COV** | **COV 2** | **ICC [95% CI]** | **n** |
|  | **Correlation (r)** |  |  |  |  |  |  | **Correlation (r)** |  |  |  |  |  |  |
| **GRS** | y=0.9932x-0.1111; P<0.0001 | 0.1±1.4 | -2.6 to 2.8 | 17.92% | 18.57% | 0.98 [0.95-0.99] | 16 | y=0.7503x+6.654; P<0.0001 | -1.418±2.82 | -6.945 to 4.109 | 22.27% | 18.84% | 0.91 [0.78 - 0.96] | 13 |
|  | r=0.9792 |  |  |  |  |  |  | r=0.9278 |  |  |  |  |  |  |
| **GCS** | y=0.9084x+3.353; P<0.0001 | -0.03±0.5 | 1.0 to 0.9 | 9.79% | 10.01% | 0.97 [0.93 -0.99] | 16 | y=0.7547x-4.117; P<0.0001 | 0.5200±1.052 | -1.542 to 2.582 | 12.90% | 11.06% | 0.89 [0.74 - 0.96] | 13 |
|  | r=0.972 |  |  |  |  |  |  | r=0. |  |  |  |  |  |  |
| **Segmental RS** | y=0.9084x+3.353; P<0.0001 | 0.2±4.4 | -8.5 to 8.8 | 33.82 | 32.81% | 0.94 [0.92 - 0.95] | 256 | y=0.6999x+8.379; P<0.0001 | -1.9±6.888 | -15.40 to 11.60 | 34.01% | 31.17% | 0.77 [0.71 - 0.82] | 207 |
|  | r=0.94 |  |  |  |  |  |  | r=0.8085 |  |  |  |  |  |  |
| **Segmental CS** | y=0.9269x-1.499; P<0.0001 | 0±1.1 | -2.2 to 2.2 | 19.80% | 19.06% | 0.96 [0.95 - 0.97] | 256 | y=0.7735x -3.801; P<0.0001 | 0.5768±2.332 | 3.993 to 5.147 | 20.07% | 19.72% | 0.77 [0.71 - 0.82] | 207 |
|  | r=0.963 |  |  |  |  |  |  | r=0.8116 |  |  |  |  |  |  |
| **GLS** | y=1.098x+1.885;  P<0.0001  r=0.938 | 0.02±0.8 | -1.5 to 1.6 | 9.84% | 11.53% | 0.96  [0.90 – 0.99] | 16 | y=0.9049x-2.295;  P<0.0001  r=0.9291 | -0.689±0.9299 | -2.511 to 1.134 | 14.79% | 13.84% | 0.95  [0.73 – 0.99] | 13 |
| **4C-GLS** | y=1.059x+1.043; P<0.0001 | 0.1±1.0 | 2.0 to 2.1 | 13.45% | 15.14% | 0.93 [0.84 - 0.97] | 16 | y=0.8557x-2.629; P=0.0006 | 0.04769±1.565 | -3.020 to 3.116 | 13.86% | 14.45% | 0.83 [0.63 - 0.93] | 13 |
|  | r=0.936 |  |  |  |  |  |  | r=0.8226 |  |  |  |  |  |  |
| **3C-GLS** | y=0.8174- 3.469; P<0.0001 | -0.1±1.3 | -2.6 to 2.4 | 14.79% | 13.53% | 0.90 [0.77 - 0.96] | 13 | y=0.7623x-3.159; P=0.0003 | 1.003±1.859 | -2.641 to 4.647 | 19.34%% | 18.65% | 0.83 [0.63 - 0.93] | 13 |
|  | r=0.899 |  |  |  |  |  |  | r=0.8388 |  |  |  |  |  |  |
| **2C-GLS** | y=0.8958x-2.091; P<0.0001 | 0.1±0.8 | -1.5 to 1.8 | 10.73% | 10.45% | 0.92 [0.81 - 0.97] | 12 | y=0.8487x-1.397; P<0.0001 | 1.148±1.357 | -1.511 to 3.808 | 18.45% | 18.66% | 0.90 [0.76 - 0.96] | 13 |
|  | r=0.914 |  |  |  |  |  |  | r=0.9003 |  |  |  |  |  |  |

SD, standard deviation; COV, coefficient of variation; ICC, intraclass correlation; CI, conf

**Figure 1. Mechanical dispersion of LV (subjects A-I).** The circumferential strain short-axis slices of the LV (y-axis) over time (cardiac cycle; x-axis) plotted on a graph for each subject included in the study. Dispersion graph for subject I is presented in figure 5 in main text.

Supplementary Video 1. A cine display presenting a sequential stack of 3D late gadolinium images acquired by IR-FLASH.

Supplementary Video 2. Cine acquisition of an apical short-axis slice displaying anterolateral regional wall motion abnormality in subject F.

Supplementary Video 3. Two chamber cine views at 15d days post-myocardial infarction in subjects J (left) and K (right), displaying left ventricular dilatation and anterior wall thinning.

Supplementary Video 4. Apical short-axis slices of subject F with circumferential strain color overlay, displaying diminished anterolateral strain.
